# Living on the edge with too many mouths to feed: Why dopamine neurons die

**DOI:** 10.1002/mds.25135

**Published:** 2012-10

**Authors:** J Paul Bolam, Eleftheria K Pissadaki

**Affiliations:** 1 Medical Research Council Anatomical Neuropharmacology Unit, Department of Pharmacology, and Oxford Parkinson’s Disease Centre,University of Oxford, Oxford, UK; 2 Department of Basic Sciences, Faculty of Medicine,University of Crete, Heraklion, Greece

**Keywords:** Parkinson’s, dopamine neurons, energy, neurodegeneration

## Abstract

Although genes, protein aggregates, environmental toxins, and other factors associated with Parkinson’s disease (PD) are widely distributed in the nervous system and affect many classes of neurons, a consistent feature of PD is the exceptional and selective vulnerability of dopamine (DA) neurons of the SNc. What is it about these neurons, among all other neurons in the brain, that makes them so susceptible in PD? We hypothesize that a major contributory factor is the unique cellular architecture of SNc DA neuron axons. Their large, complex axonal arbour puts them under such a tight energy budget that it makes them particularly susceptible to factors that contribute to cell death, including unique molecular characteristics associated with SNc DA neurons and nonspecific, nervous-system–wide factors. © 2012 Movement Disorder Society

The causes of Parkinson’s disease (PD) are varied and complex and include a variety of genetic and environmental factors, but the majority of cases are idiopathic. Selective degeneration of dopamine (DA) neurons of the SNc is responsible for the principal motor symptoms of PD. Factors that contribute to their cell death are many and varied and include protein mishandling, oxidative stress, inflammation, calcium dynamics, toxicity of DA metabolites, and, frequently, mitochondrial dysfunction.1–12 The loss of neurons in PD is, however, not confined to the substantia nigra. It has long been known that cell death occurs at many levels of the nervous system; for instance, there is a marked loss of cholinergic neurons in the pedunculopontine nucleus^13^ and noradrenergic neurons in the locus coeruleus.^14^ Indeed, cell death is associated with neurons that possess long axons that are unmyelinated in their region of aborization at least.^15^, ^16^ Furthermore, it has recently been proposed that PD is a dynamic, widespread disease. Lewy body pathology and cell death is first identified in the brain stem (or possibly even the enteric nervous system) and there is a slow progression to the forebrain; when the wave of pathology reaches the midbrain DA neurons, then the cardinal motor symptoms of PD are expressed.^15^, ^16^

Factors considered to contribute to cell death in PD (see above) are also found widely distributed in the nervous system,1–3 but the consistent feature of both idiopathic and genetic forms of PD is the selective, and exceptional, vulnerability of DA neurons of the SNc.^3^ This is one of the most critical issues in the field of PD research: Why is it that DA neurons of the SNc, above all other neurons in the brain, and indeed above other classes of DA neuron, show such selective vulnerability in PD and in models of PD? The explanation is relatively straightforward when one considers toxins such as 6-hydroxydopamine and MPTP and its active metabolite, 1-methyl-4-phenylpyridinium (MPP^+^), both of which are used to create animal models of PD.^17^ Their selectivity for DA neurons is because their access to the intracellular compartment is dependent on the DA transporter, which is only expressed by DA neurons and, interestingly, at lower levels in DA neurons of the ventral tegmental area (VTA), which are less susceptible to toxins and PD.^19^ However, the explanation is more complex when one considers general mitochondrial poisons, such as rotenone (mitochondrial complex I inhibitor), which, when used at appropriate doses, leads to selective, dramatic loss of DA neurons in the SNc.20–22 Similarly, when one considers genetic variants of PD, it is usually the case that the mutated proteins are expressed widely in the brain (e.g., LRRK2^1^, ^2^, ^4^, ^5^ and do not show selectivity for DA neurons). Furthermore, in idiopathic PD (iPD), mitochondrial dysfunction that has been identified postmortem in the substantia nigra is not confined to this area, but is evident in many brain regions and in other tissues, including skeletal muscle and platelets.^2^ Why, in these situations, is it that DA neurons of the SNc show such selective vulnerability?

In this article, we will not address the molecular characteristics of SNc DA neurons that have been proposed to contribute to their selective vulnerability (see reviews above), but we suggest that a major contributory factor that underlies their selective vulnerability is their uniquely massive, unmyelinated axonal arbor. Axons of DA neurons of the SNc give rise to a massive number of synapses and a massive axonal field.23–25 The extreme bioenergetic demands of maintaining these unique morphological features and of maintaining neuronal signaling puts SNc DA neurons energetically “on the edge”—they are at a marked disadvantage in that they simply have “too many mouths to feed,” thus making them especially vulnerable to stressors of any sort, including environmental and genetic factors, whose effects are widely distributed in the body, and factors that are specific to DA neurons.

## Axonal Arbor of DA Neurons

It is well recognized that DA neurons provide a massive, and importantly unmyelinated, innervation of the forebrain, a fact that was evident even in the earliest histofluorescence studies of DA neurons^26^ and, by analysis of sections of the forebrain (of any mammalian species) stained to reveal markers of DA neurons (e.g., tyrosine hydroxylase; see, for instance, Björklund and Lindvall^27^), the synthetic enzyme of catecholamines. Quantitative analyses of the numbers of DA neurons in the midbrain and their innervation of the forebrain in rats have revealed the true nature of this innervation^23^, ^27^, ^28^ (Table 1). Rat SNc contains approximately 12,000 DA neurons,^29^, ^30^ which provide the dense innervation of the striatum. Because we know the number of neurons in the striatum and SNc in rats, as well as quantitative details about the precise synaptic relationship between dopaminergic axons and the principal neuron of the striatum, the medium spiny neuron (MSN),^23^ we can estimate that, in rats, the axon of a single SNc DA neuron gives rise to between 102,165 and 245,103 synapses (Table 1) at the level of the striatum. Our estimate is in the same order of magnitude as other studies in rats; estimates of the number of axonal varicosities (putative synapses) of a single DA neuron by Andén et al.^26^ (250,000 varicosities per neuron; recalculated by Björklund and Lindvall^27^) and the estimate of the number of synapses by Wickens and Arbuthnott (369,881 synapses per neuron^28^) that was based on the density of synapses in the striatum.

**Table 1:** Estimation of the number of synapses formed by a single DA neuron with MSNs in the rat striatum

1. Average number of dendritic spines on one MSN	6,250 to 15,000^56^
2. Percentage of axospinous synapses that involve cortical and thalamic terminals	64.8%^57^^a^
3. Average number of dendritic spines postsynaptic to cortical or thalamic terminals on one MSN (value 1) × (value 2)	4,050 to 9,720
4. Proportion of these dendritic spines in synaptic contact with a DA terminal	0.06666^23^
5. Number of these dendritic spines on one MSN in synaptic contact with a dopamine terminal (value 3) × (value 4)	270 to 648
6. Percentage of DA terminals that contact dendritic spines (not shafts or cell bodies)	61.3%^23^
7. Multiplying factor to incorporate synapses with dendritic shafts and cell bodies; reciprocal of 0.613 (value 6)	1.632
8. Number of DA terminals forming synapses with one MSN (value 5) × (value 7)	441 to 1,058
9. Number of MSNs in the striatum (one hemisphere)	2,780,000^30^
10. Number of DA terminals forming synapses with all MSNs in one hemisphere (value 8) × (value 9)	1,225,980,000 to 2,941,240,000
11. Number of DA neurons in the SNc (one hemisphere) of rat	12,000^29^
12. Number of synapses formed by one DA neuron in the rat striatum (value 10)/(value 11)	102,165 to 245,103

Note that to arrive at the number of dopaminergic synapses in contact with an individual MSN, quantitative data from spines that are postsynaptic to cortical or thalamic terminals was used.^23^ This will introduce error (i.e., an underestimate of the number of synapses) because dopaminergic terminals may contact other spines. Table is modified from Moss and Bolam.^24^

^a^This figure represents the percentage of axospinous synapses involving VGluT1-positive (cortical) and VGluT2-positive (thalamic) terminals. It is likely to be an underestimate as a result of false-negative labeling of terminals.

The figure of hundreds of thousands of synapses can be put into perspective when one considers the axons of other types of neurons in the basal ganglia of the rat. Based on the analysis of single cells and counts of axonal varicosities, the principal type of neuron in the globus pallidus (external) gives rise to approximately 2,000 synapses, an MSN gives rise to 300–500 synapses, and a fast-spiking GABA interneuron in the striatum gives rise to approximately 5,000 synapses.31–35 Thus, SNc DA neurons possess at least two orders of magnitude more synapses than other neurons in the basal ganglia.

Estimates of such a large number of synapses of SNc dopaminergic neurons are consistent with the findings of Matsuda et al.^25^ These investigators used a viral vector based upon a Sindbis virus designed to express green fluorescent protein that labels infected neurons in a Golgi-like manner,36–38 an approach that is necessary to get good labeling of dopaminergic axons. They demonstrated that the axons of single DA neurons of the SNc labeled *in vivo* in the rat occupy up to approximately 6% of the volume of the striatum, with a total length of all the axonal branches of up to 78 cm (average total length = 46.6 cm) that branches frequently (mean interbranch interval of ∼31 μm) and gives rise to a highly tortuous axonal arbor. Note that this figure is remarkably close to the estimate of 30 cm, based on the analysis of histofluorescently labeled axons in the striatum (Andén el al.^26^; recalculated by Björklund and Lindvall^27^). It should be noted that the observations of Matsusda et al.^25^ reveal much larger axonal arbors than reported on by Prensa and Parent,^39^ who used extracellular deposits of biotinylated dextran amine (BDA) to label axons. Although this study provided critical qualitative information about the collateralization of the axons of DA neurons, the sizes of the axonal fields were not quantified. Calculations, based on representations of the BDA-labeled axons and estimates of the number of synapses/varicosities formed by the axon of an individual SNc dopaminergic neuron, suggest that the use of nonreplicating markers leads to underestimates of the size of axonal arbors (see Supporting Materials).

What are the likely consequences of such an extensive unmyelinated axonal field and large number of synapses? One can predict that such a large neuronal architecture would put the neuron under a phenomenal energy demand not just to maintain healthy cell biological functions (e.g., protein synthesis, cytoskeleton maintenance, axonal transport, and so forth), but also in the maintenance of the membrane potential, the propagation of action potentials, and in synaptic transmission. These neuron-specific aspects of cell function are energetically expensive, particularly if the axon is not myelinated,^40^ as is the case for DA neurons. We propose that the tight energy budget that this unique axonal architecture imposes on SNc DA neurons is a critical factor in their susceptibility in PD and animal models of PD. They are under a bioenergetic demand that is so extreme that they are energetically on the edge. Under normal circumstances, there is no effect of this high energy demand on the neurons. However, any situation that perturbs the balance between energy production and demand—such as mitochondrial dysfunction or oxidative stress—would “tip them over the edge,” such that energy demand exceeds supply. A negative energy balance would lead to many detrimental consequences within a neuron, including further oxidative stress, further mitochondrial dysfunction, inability to deal with protein turnover, impaired autophagy, and so on, all factors considered to contribute to cell death in PD (for references, see above). A negative energy balance would thus lead to functional failure and, eventually, cell death.

Any theory that accounts for the loss of SNc DA neurons in PD must include an explanation as to why DA neurons of the VTA, or indeed calbindin-positive SNc DA neurons, are relatively spared in PD or models of PD.^13^, ^41^ It is clear that they have different molecular and physiological characteristics that contribute to the differential susceptibility^3^, ^19^, ^42^; however, they also have strikingly different morphological characteristics. In contrast to DA neurons of the SNc, DA neurons of the VTA are likely to give rise to far fewer synapses. We estimate that a DA neuron in the VTA of rats gives rise to 12,000 to 30,000 synapses.^24^ This figure is based on the fact that approximately twice as many DA neurons in the VTA^29^ innervate the ventral striatum, which has a volume of approximately one fifth of that of the dorsal striatum (Table 2). This estimate assumes that the pattern and density of innervation of the ventral striatum (nucleus accumbens) by VTA neurons is similar to the pattern and density of innervation of the dorsal striatum by SNc DA neurons. Thus, those DA neurons that are less susceptible in PD in humans and in animal models of PD have an order of magnitude fewer synapses and much smaller axonal arbors and thus a bioenergetic demand to maintain their structural and functional properties that is far less than that of SNc DA neurons. We do not have an estimate of the number of synapses formed by calbindin-positive DA neurons in the SNc that also innervate the striatum, but our hypothesis predicts that they give rise to a far smaller number of synapses.

**Table 2:** Estimation of the number of synapses formed by a single VTA DA neuron in the ventral striatum of the rat

Number of DA neurons in the SNc (one hemisphere)	12,000^29^
Volume of striatum (mm^3^)	19.9^30^
Volume of (dorsal) striatum per SNc DA neuron (mm^3^)	0.001658 (19.9/12,000)
Number of DA neurons in the VTA (one hemisphere)	20,000^29^
Volume of ventral striatum (mm^3^) (approximately one fifth of size of striatum)	∼ 4 (19.9/5)
Volume of ventral striatum per VTA DA neuron (mm^3^)	0.0002 (4/20,000)
Ratio of ventral versus dorsal striatum volume per DA neuron	0.121 (0.0002/0.001658)
Number of synapses formed by one DA neuron in the ventral striatum	12,351 to 29,644 (0.121 × 102,165^a^) to (0.121 × 245,103^a^)

^a^Estimated number of synapses formed by one SNc DA neuron in the striatum in the rat (see Table 1).

## Characteristics of Axons of Human DA Neurons

All of the detailed quantitative analyses of the axons of DA neurons have been carried out in rats; however, rats do not spontaneously develop PD, whereas humans do. There are a number of explanations for this. One of the strongest risk factors for PD is age. However, comparative anatomy and quantitative analyses reveal that the bioenergetic demand on SNc DA neurons in humans is likely to be much greater than in rats (Table 3). The volume of the striatum has increased by approximately 300-fold from rats (19.9 mm^3^) to humans (6,280 mm^3^),^30^, ^43^ but the number of DA neurons in the SNc has increased by only 32-fold (rats, 12,000; humans, 382,000).^29^, ^44^ If we assume that the density and pattern of innervation of the striatum by DA neurons in humans is similar to that in rats, then an individual SNc DA neuron in humans should give rise to 10 times the number of synapses in rats—that is, a single SNc DA neuron in humans is estimated to give rise to between 1 and 2.4 million synapses at the level of the striatum (Table 3). Furthermore, if the branching patterns of DA neuron axons are similar in rats and humans, then we can estimate that the average total length of the axon of an individual SNc DA neuron is in the region of 4.5 meters with—assuming a similar interbranch interval to that in rats—an arborization of incredible complexity. Thus, the bioenergetic demand to maintain the structure and functionality of SNc DA neurons in humans is likely to be an order of magnitude greater than that required for rat SNc DA neurons. This, together with longevity, may account for why humans, and not rats, get PD.

**Table 3:** Estimation of the number of synapses formed by a single DA neuron in the striatum in humans

Number of DA in the SNc (one hemisphere)	382,000^44^
Volume of striatum (mm^3^)	6,280^43^
Volume of striatum per SNc DA neuron (mm^3^)	0.01644 (6,280/382,000)
Ratio human versus rat of striatum volume per DA neuron	9.916 (0.01644 /0.001658^a^)
Number of synapses formed by one DA neuron in the human striatum	1,013,068 to 2,430,441 (9.916 × 102,165^b^) to (9.916 × 245,103^b^)

^a^Volume of striatum per SNc DA neuron in rat (see Table 1).

^b^ Estimated number of synapses formed by one SNc DA neuron in the striatum in the rat (see Table 1).

It should be noted that although the innervation of the striatum by DA axons far exceeds that in other regions of the brain, our estimates of numbers of synapses do not take into account the innervation of extrastriatal regions by SNc or VTA neurons nor the differences in the density of innervation of these regions across species.^45^, ^46^ Although this is likely to lead to underestimates of the numbers of synapses, the orders of magnitude are likely to be similar.

We have begun to address the hypothesis by the creation of a biology-based computational model of the axons of DA neurons. Data derived from the model suggests that the energy cost of the propagation of action potentials and recovery of membrane potential grows as a power law with respect to the size and complexity of the axonal arbor.^47^ Thus, DA neurons of the SNc not only have disproportionately large, complex axonal arbors, compared with other dopamine neurons and other neurons in the brain, but the very nature of the size and complexity means that the energy cost of signal propagation is disproportionately greater.

## Implications for Treatment and Understanding of PD

What are the implications of this hypothesis for PD and its treatment? The idea put forward in this article suggests that DA neuron death in iPD, and probably other forms of PD, is ultimately a consequence of energy supply—iPD is not specific for DA neurons, it is just that SNc DA show the greatest susceptibility when energy metabolism is perturbed, whether the cause be a specific deficit in DA neurons or a nonspecific insult.^48^ This is not a new idea in itself, because it is well recognized that mitochondrial dysfunction is associated with idiopathic and other forms of PD.^2^ Indeed, it has recently been proposed by Wellstead and Cloutier, on the basis of a systems biology approach, that PD is a disease of energy metabolism.^49^ Furthermore, mitochondria in DA axons are smaller and transported more slowly than other neurons,^50^ and consistent with the hypothesis, it has been proposed that axons preferentially degenerate in PD.^51^ Our hypothesis, however, offers an explanation as to why it is that SNc DA neurons are particularly susceptible to impaired energy metabolism and/or supply.

One might suggest that a novel approach to the treatment of PD could be to enhance energy supply to DA neurons, whether this be by genetic or molecular means. The use of growth factors^52^ at the level of the striatum, which promote the growth and “sprouting” of the axons of surviving DA neurons,^53^, ^54^ may be counterproductive, in that it would increase the energy demand on individual neurons and hence their susceptibility. Because the sprouting of the axons of surviving DA neurons occurs once degeneration has begun, perhaps cell-based therapies, such as deposits of fetal DA neurons or, in the future, stem cells should be initiated early in the progression of the disease before such sprouting has occurred.

Whatever the implications for disease treatment, we suggest that the unique axonal architectural features and unmyelinated nature of the axons of DA neurons of the SNc, especially in humans, render these neurons particularly sensitive to DA-neuron–specific and –nonspecific stressors. This suggests that iPD is a nonspecific disease—it is simply the case that the very nature of the structure of SNc DA neurons means that these neurons fall into a negative energy balance, and ultimately die, as a consequence of longevity coinciding with DA-neuron–specific and/or –nonspecific stressors in the nervous system. Thus, the Achilles heel of SNc DA neurons^55^ includes not only their unique molecular characteristics, but also their unique morphological features.
