# Supplementary material for: Living on the edge with too many mouths to feed: Why dopamine neurons die
Source: Mov Disord. 2012 Oct;27(12):1478–83. doi: 10.1002/mds.25135 (PMC3504389; doi:10.1002/mds.25135)
Supplement: Supplementary file 1 [file mds0027-1478-SD1.doc]

**MDS-12-0502.R3**

**Bolam and Pissadaki: Supplementary information.**

In a study of axons labelled by extracellular deposits of biotinylated dextran amine (BDA), Prensa and Parent described many unique anatomical features of nigrostriatal axons. Their data provided important information about the degree of collateralization to nuclei other than the striatum and the relationship of the axonal arbours to the patch/matrix organisation of the striatum. However, the size of the axonal field and volume of striatum occupied by individual axons as revealed in their figures appears far smaller than those of Matsuda et al who used a replicating viral vector (with a GFP reporter) to label individual axons. Although, Prensa and Parent did not provide any quantification of their labelled axons, and indeed this was not the objective of their study, Matsuda and colleagues made estimates from their figures (see supplementary material in ). Their measurements suggest that the mean total axonal length of individual nigrostriatal axons labelled by the BDA is 36,193 µm as opposed to 466,831 µm labelled by the viral vector. One possibility for this discrepancy is that the viral constructs used by Matsuda and colleagues leads to sprouting of axons. However, in view of the relatively short survival times, the fact that other studies using the same approach reveals axonal arbours consistent with what has been predicted previously and our estimates of synapse/varicosity density (see below), this is unlikely.

Three independent estimates of the number of synapses/varicosities arising from a single SNc dopaminergic axon using different approaches and different assumptions, have been used to estimate the density of synapses/varicosities (see Suppl Table1). Applying these to the estimate of the total length of dopaminergic axons labelled by BDA, suggests a density of 3.4-12 synapses/varicosities per µm whereas the data from viral vector labelling suggests a density of 0.2-0.8 synapses/varicosities per µm. Since a varicosity or synaptic bouton is in the order of 0.5 µm in diameter, it is clear that the estimates of total length derived from the use of a viral vector is more realistic. We can only conclude that the relatively small axonal fields observed when using standard anterograde markers (e.g. biotinylated markers, peroxidase conjugates, or *Phaseolus vulgaris* leucoagglutinin) is probably a consequence of the failure to take up sufficient marker to label the whole axonal field, only when a replicating viral vector designed to express a marker is used, can the level of the marker be high enough to label the whole of the axonal field.

**Supplementary Table 1: Estimates of the density of synapses/varicosities along dopaminergic axons**

Mean total axon length revealed by viral vectors : 466,831 µm*

Mean total axon length revealed by BDA : 36,193 µm *

Number of synapses per DA neuron reported by Wickens & Arbuthnott : 369,881

Number of synapses per DA neuron reported in this paper: 102,165 to 245,103

Number of varicosities per DA neuron reported by Anden : 250,000

***Density of synapses/varicosities along the axons of dopamine neurons revealed by BDA.***

Density of synapses from Wickens & Arbuthnott data is given by: 369,881/30,193 synapses per µm  **12 synapses** **per µm**

Density of synapses from our data is given by: 102,165/30,193 and 245,103/30,193 synapses per µm  **3.4 - 8.1 synapses per µm**

Density of varicosities from Anden data is given by: 250,000/30,193  **8.3 varicosities per µm**

***Density of synapses/varicosities along the axons of dopamine neurons revealed by viral vector.***

Density of synapses from Wickens & Arbuthnott data is given by: 369,881/460,831 synapses per µm  **0.8 synapses** **per µm**

Density of synapses from our data is given by: 102,165/460,831 and 245,103/460,831 synapses per µm **0.22 - 0.53 synapses per µm**

Density of varicosities from Anden data is given by: 250,000/460,831 **0.54 varicosities per µm**

Estimates of Wickens & Arbuthnott are based on estimates of the numbers of synapses in the striatum and number of neurons in striatum and SNC

Estimates reported in the present paper are based on the synaptic organisation of the striatum and number of neurons in striatum and SNC

Estimates of Anden et al are based on the density of histofluorescent profiles in the striatum and number of neurons in SNC.

* For calculations of density, 6,000 µm is subtracted from the total length of the axon to account for that portion of the axon before it enters striatum (i.e. where synapses are not formed).

DA, dopamine; BDA, biotinylated dextran amine

**References**

1. Prensa L, Parent A. The nigrostriatal pathway in the rat: a single-axon study of the relationship between dorsal and ventral tier nigral neurons and the striosome/matrix striatal compartments. J Neurosci 2001; 21:7247-7260.

2. Matsuda W, Furuta T, Nakamura KC, Hioki H, Fujiyama F, Arai R, Kaneko T. Single nigrostriatal dopaminergic neurons form widely spread and highly dense axonal arborizations in the neostriatum. J Neurosci 2009; 29:444-453.

3. Fujiyama F, Sohn J, Nakano T, Furuta T, Nakamura KC, Matsuda W, Kaneko T. Exclusive and common targets of neostriatofugal projections of rat striosome neurons: a single neuron-tracing study using a viral vector. Eur J Neurosci 2011; 33:668-677.

4. Ohno S, Kuramoto E, Furuta T, Hioki H, Tanaka YR, Fujiyama F, Sonomura T, Uemura M, Sugiyama K, Kaneko T. A morphological analysis of thalamocortical axon fibers of rat posterior thalamic nuclei: A single neuron tracing study with viral vectors. Cerebral Cortex 2011; doi: 10.1093/cercor/bhr356

5. Wickens J, Arbuthnott GW Strutural and functional interactions in the striatum at the receptor level. In: Dunnett SB, Bentivoglio M, Björklund A, Hökfelt T. Eds. Handbook of Chemical Neuroanatomy 2005 21:199-236, Elsevier.

6. Andén NE, Fuxe K, Hamberger B, Hökfelt T. A quantitative study on the nigro-neostriatal dopamine neuron system in the rat. Acta Physiol Scand 1966; 67:306-312.

7. Björklund A, Lindvall O Dopamine-containing sytems in the CNS. In: Björklund A, Hökfelt T. Eds. Handbook of Chemical Neuroanatomy 1984; 2:55-122, Elsevier.
